# Gut Bacteriophage: Current Understanding and Challenges

**DOI:** 10.3389/fendo.2019.00784

**Published:** 2019-11-29

**Authors:** Thomas D. S. Sutton, Colin Hill

**Affiliations:** APC Microbiome Ireland and School of Microbiology, University College Cork, Cork, Ireland

**Keywords:** virome, bacteriophage, microbiome, phage-host interactions, microbiota

## Abstract

The gut microbiome is widely accepted to have a significant impact on human health yet, despite years of research on this complex ecosystem, the contributions of different forces driving microbial population structure remain to be fully elucidated. The viral component of the human gut microbiome is dominated by bacteriophage, which are known to play crucial roles in shaping microbial composition, driving bacterial diversity, and facilitating horizontal gene transfer. Bacteriophage are also one of the most poorly understood components of the human gut microbiome, with the vast majority of viral sequences sharing little to no homology to reference databases. If we are to understand the dynamics of bacteriophage populations, their interaction with the human microbiome and ultimately their influence on human health, we will depend heavily on sequence based approaches and *in silico* tools. This is complicated by the fact that, as with any research field in its infancy, methods of analyses vary and this can impede our ability to compare the outputs of different studies. Here, we discuss the major findings to date regarding the human virome and reflect on our current understanding of how gut bacteriophage shape the microbiome. We consider whether or not the virome field is built on unstable foundations and if so, how can we provide a solid basis for future experimentation. The virome is a challenging yet crucial piece of the human microbiome puzzle. In order to develop our understanding, we will discuss the need to underpin future studies with robust research methods and suggest some solutions to existing challenges.

## Introduction

The human gastrointestinal tract (GIT) is a complex environment containing billions of microorganisms (1). Changes in oxygen concentration, pH, nutrient availability, water availability and bile salts shape the relative abundance of microorganisms from all domains of life (fungi, protists, bacteria, and archaea) (2–5). Of these microorganisms, bacteria are by far the most characterized, making up the vast majority of the DNA sequences and biomass (6, 7). This bacterial community also plays a central role in normal physiology of the mammalian gut by facilitating metabolic functions, protecting against pathogens and modulating the immune system (8–10). Similarly, alterations in the composition and abundance of this bacterial community are closely associated with diseases such as irritable bowel syndrome (IBS), inflammatory bowel disease (IBD), colorectal cancer (CRC), *Clostridium difficile* infection (CDI), obesity, and neurological disorders (11–15). However, the forces that shape the composition of these bacterial communities remain poorly understood and this has slowed the development of microbiome based therapeutics and biomarkers.

Bacteriophage (phage) are viruses that infect prokaryotic hosts and play crucial roles in shaping the composition and diversity of bacterial communities in many environments, facilitating horizontal gene transfer, and nutrient turnover through continuous cycles of predation and coevolution (16–18). To date, the majority of viral metagenome (virome) research has been focused on environmental communities such as those in the ocean (19, 20). In this environment, the virome is central to the movement of dissolved organic matter across trophic levels of the ocean food chain and between the surface and the depths of the water column (16, 21). A growing body of evidence also suggests the virome can shape the functional capacity of host communities encoding functions such as photosynthetic genes in the photic zones of the ocean (22) and bacterial virulence factors in pathogenic bacteria (23).

Phage make up the vast majority of the viral component of the gut microbiome (24). They are also believed to play a key role in shaping the composition and function of the human gut microbiome in both health and disease (25–27). However, despite being highly abundant in the gut (>10^10^ g^−1^) (28, 29) and having considerable impacts on microbial ecosystems, they remain one of the least understood members of the gut microbiome. Early sequencing studies of the human gut virome estimated that it was dominated by novel sequences, with only 41% sharing homology to databases (30). However, as sequencing platforms and library preparation methods improved and yielded a more detailed view of the virome, this unknown majority or “viral dark matter” was found to make up an even greater proportion of the virome, lowering the identifiable fraction to as little as (1–14%) (31).

Since phage were first identified by Frederick Twort in 1915 (32), culture-based methods such as plaque assays have been used to screen and quantify phage titers from many environments. Today, these methods still play a central role in identifying phage which target specific bacteria and have contributed to our understanding the mechanics of phage host interactions and replication cycles. However, as the vast majority of phage-host pairs in the gut are unknown, these methods are not suited to large-scale characterization of a complex ecosystem such as the human gut. Additionally, many of bacteria in the human gut are not routinely cultivated, despite recent advances (33). As a result, virome studies lean heavily on sequencing based metagenomic approaches to investigate gut phage communities and to try to understand their role in shaping the gut microbiome (31). This involves sequencing the total viral DNA and RNA from a community following physical separation from the bacterial component, using assembly software to recreate the viral genomes within that community and characterizing abundance and function of those genomes. However, many sequence-based virome studies exclude viral dark matter from analysis, working largely with a small fraction of known phage sequences (usually 1–14% of the dataset). This can have profound implications for the conclusions drawn from these studies, as changes in the known fraction may not reflect changes in the virome as a whole. As a result, database-independent analysis methods are increasingly being used which include both known and unknown fractions of the virome (29). However, high levels of inter-individuality make biological signals across virome studies difficult to detect (24, 34, 35). Furthermore, virome studies are particularly susceptible to methodological bias due to difficulties in benchmarking *de novo* bioinformatic tools and the dominance of unknown sequences in virome datasets (36–38).

We will discuss phage of the human gut virome and their interactions with the microbiome. We will also highlight how little we know about their role in human health. Finally, we will discuss critical areas of virome analysis methods which must be addressed and improved upon if we are to fully understand the role of phage in shaping the microbiome and human health.

## How Phage Interact With Bacterial Hosts

### Phage Infection Cycles

**Figure 1:**
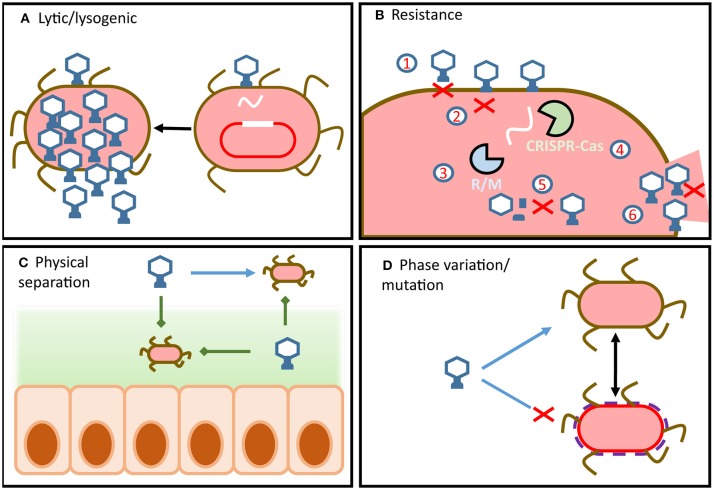
Overview of phage-host dynamics in the gut. **(A)** Phage infection can lead to virulent or temperate replication cycles. Integrated temperate phage use internal and external signals from hosts to determine if or when to enter the lytic cycle. **(B)** Bacteria can possess a wide array of defense mechanisms which target different steps of the phage replication cycle. Similarly, phage encode a wide array of counter-defense mechanisms which target host defenses and allow the phage to remain infectious. **(C)** Physical separation of phage and host (e.g., in mucous or in lumen) means that dynamics change along the radial and longitudinal axes of the gut. **(D)** Strain-level variation can result from resistance by mutation or by phase variation.

As obligate parasites of bacteria, phage persistence in a microbial ecosystem is dependent on the presence of a suitable sensitive host. Phage infection is typically followed by one of two replication cycles, lytic or lysogenic (39) (Figure 1A). In both cases a phage virion binds to the host cell surface using a phage receptor-binding protein triggering the insertion of its genome into the host. For lytic phage, subsequent translation of the phage genetic material by the host cell results in the replication of the phage genome, assembly of phage particles and lysis of the host. This results in the release of new phage virions into the environment that can infect nearby hosts. Alternatively, lysogenic infection results in the replication of the phage genome within the host cell without the immediate synthesis of phage virions. The phage genome may integrate into that of the host where it exists as a prophage, replicating together with the host genome and thus persisting in resulting daughter cells. In the case of pseudolysogeny, the phage genome persists as an episome within the host cell, separate to the host genome. In order to ensure subsequent daughter cells contain phage genomes pseudolysogenic phage can use maintenance systems such as toxin-antitoxin (40, 41). However, cases of daughter cells lacking pseudolysogenic phage have also been reported (41). Following an induction event, the lysogenic phage will initiate the translation of its genome and subsequent production of phage virions leading to host lysis. Additionally, phage such as M13 undergo chronic non-lethal infection cycles, where newly produced virions exit the cell without lysis (42). However, little is known about the prevalence of these different lifecycles in the human gut.

### Resistance and Counter-Resistance

Bacterial hosts employ a wide array of phage resistance mechanisms which have been comprehensively reviewed by Labrie et al. (43) and Rostøl et al. (44). To prevent phage adsorption, bacterial cells can differentially express or mutate cell surface receptors (Figure 1B1) (45, 46), S-layer proteins (47), or produce protective cell surface polysaccharides (48). Additionally, bacterial hosts can reduce the numbers of phage particles available to infect hosts by producing outer membrane vesicles (49). These bind and sequester phage particles, reducing their numbers in the environment and thereby the risk of infection. Should the phage successfully bind to the appropriate surface receptor, the fate of the host is not yet sealed as anti-phage resistance mechanisms extend to all steps of the phage infection cycle.

Hosts can prevent phage DNA injection entirely by modifying inner-membrane proteins (Figure 1B2) (50), identify and degrade injected phage DNA using restriction modification systems (Figure 1B3) (51) and CRISPR-Cas systems (Figure 1B4) (44), chemically block phage DNA replication (52), or prevent virion assembly (Figure 1B5) (53).

Should these defense mechanisms fail to prevent phage replication within the cell, the bacterial host can sacrifice itself in order to protect its sister cells (54). These are referred to as abortive infection systems and act by shutting down cellular functions to prevent phage release (Figure 1B6).

Phage defense systems are regularly encoded on mobile genetic elements that can facilitate the transfer of resistance across the bacterial community. However, due to their metabolic costs bacterial cells rarely encode more than one of these systems (55). This gives rise to complex dynamics between hosts with the metabolic burden of resistance and susceptible hosts with fitness advantages. In addition, a cell carrying a prophage can be made resistant to other phage in what is referred to as superinfection exclusion (56). To further complicate these relationships, antagonistic coevolution of phage-host pairs has led to the development of phage counter-resistance mechanisms, which allow phage to remain infectious in the face of a resistant host population (57). Phage counter-resistance can range from glycosidases to degrade host capsules (58) and reveal binding sites, to directed mutagenesis or hypervariable receptor binding proteins (59–61). These systems allow phage to retain compatibility with modified host receptors and also allow for the expansion of host range. Phage can even overcome host CRISPR-Cas by mutating or deleting CRISPR target sites or expressing anti-CRISPR proteins to directly interfere with CRISPR-Cas activity (62). Some of the most striking phage counter-resistance mechanisms include alteration of phage DNA to evade host restriction modification mechanisms (63) and phage encoded CRISPR-Cas systems that target and disable a range of host defense systems (64).

### Ecological Relevance of Phage-Host Dynamics

These mechanisms of infection, resistance and counter-resistance underpin virome-microbiome interactions. Thus, in order to investigate how the virome shapes or reflects the microbiome, we must first understand these interactions in the GIT. Understanding phage-host interactions is also vital if we are to use the virome as a diagnostic or therapeutic tool in the future. To this end, numerous ecological models have been used to describe phage-host interactions in the context of a biological system. Some models focus entirely on the interplay of resistance and infectivity such as the arms race dynamics model (65). This model proposes that phage infection applies selective evolutionary pressure for mutations in the hosts, resulting in resistant host populations. These mutations in turn select for phage mutations that restore infectivity, resulting in predator prey cycles (66). Other models take into account phage-host density and the metabolic cost of resistance such as the fluctuating-selection dynamics model (67, 68). This proposes that as phage predation selects for resistant hosts, it will also reduce the number of phage virions in the environment. This absence allows for the expansion of susceptible bacterial strains which lack the metabolic burden of resistance and therefore out-compete resistant hosts in the absence of phage. This transient resistance and infectivity of phage-host communities results in short-term fluctuations of phage and host numbers, but the long-term persistence of both (69).

It is important to note that strain-level fluctuations of phage-host are difficult to study in the context of the microbiome, making it difficult to verify or quantify this model. Strain-level variation within bacterial hosts cannot be detected by 16S rDNA analysis (67) and hampers metagenomic assembly of phage (38, 70). Despite these challenges, recent insights have proposed that variation in capsular polysaccharides encoded by the abundant gut bacterium *Bacteroides thetaiotaomicron* play a central role in phage susceptibility (71). This mechanism supports the concept of fluctuating-selection dynamics, as phase variation of the capsular polysaccharides creates heterogeneous host phenotypes within an isogenic population. This in turn leads to transient phage resistance across the population as host phenotypes are dynamic and non-uniform (72). Additionally, this resistance can occur without the need for horizontally-transferred resistance or mutation (Figure 1D).

Phage replication not only requires the host to be present and susceptible but it must also be metabolically active. It has therefore been proposed that the metabolic state of the host is one of the primary barriers to phage infection (69). In this way bacterial hosts can be transiently resistant to phage infection due to a lack of nutrient availability and a dormant growth phase, without incurring the metabolic cost of encoding resistance (Figure 1D) (73). Additionally, this mechanism would support the proliferation of both phage and host in the GIT, independent of resistance/counter-resistance dynamics. Nutrient availability varies significantly along the GIT, which implies that phage-host dynamics in the proximal or distal colon may be significantly different to those in fecal samples (Figure 1C) (74, 75). As the majority of virome studies draw conclusions from fecal samples, our current understanding of phage host dynamics in the GIT is limited.

The kill-the-winner ecological model is an extension of lotka-volterra dynamics applied to phage-host interactions. This model describes rapid changes of diversity and abundance of both phage and their hosts. As the most abundant bacteria are killed by their phages, other bacterial taxa will take over the ecological niche and be subsequently killed by their phages. In this way high levels of phage-host diversity and abundance are maintained (76). In ecosystems where lotka-volterra or kill-the-winner dynamics can be applied, phage exhibit an exclusively predatory relationship on hosts and the microbial biomass is significantly below the carrying capacity of the ecosystem (77, 78). However, in the human gut ecosystem microbial biomass approaches the carrying capacity of the ecosystem and the virus to microbe ratio (VMR) is low (79). Despite reports of kill-the-winner dynamics in infants (80, 81), this suggests that these models cannot not fully explain phage-host interactions in the healthy adult gut (76). Additionally, these dynamics overlook lysogeny and conditions that govern the switch between lytic and lysogenic replication cycles. Furthermore, it has been proposed that the gut virome is dominated by temperate phage (82) and that compositional changes in temperate phage communities are associated with disease states (25). Consequently the mechanisms that determine the switch between lytic and lysogenic replication cycles are also central to understanding virome-microbiome dynamics. Recent reports have described phage which can hijack bacterial quorum sensing machinery to determine the density and metabolic activity of the bacterial population from within the host cell (83). This in turn, could dictate whether persisting within in the host genome or excising and entering the lytic cycle would favor phage proliferation (Figure 1A). In addition, single cell analysis of phage-host interactions between the temperate P22 phage and *Salmonella typhimurium* suggested that phage were directly involved in creating transiently resistant host subpopulations (41). This allowed for both lysogenic and lytic replication without impeding host proliferation. Despite these intriguing insights, little is known about the dynamics of temperate to lytic switching in the mammalian GIT, highlighting a crucial target for future virome research.

Ecological models that consider the switch between lytic and lysogenic replication such as the piggyback-the-winner model appear to support experimental evidence of phage-host dynamics within the mammalian GIT (84, 85). This model focuses on a lytic to lysogenic switch that is host density dependent. Traditionally phage were believed to enter the lysogenic cycle in cases of high VMR (i.e. increased phage abundance, decreased host abundance) as a means to persist in the environment until host density can support lytic replication cycles. However, experimental evidence from coral reef ecosystems suggested that phage also entered the lysogenic cycle in high host density situations (84). Subsequently, phage can “piggyback” on host success in the ecosystem at that particular time. This model has also been proposed for phage-host interactions on mucosal surfaces in the GIT. It was suggested that at the mucosal surface, high bacterial colonization and high VMR gives rise to piggyback the winner dynamics, whereas deeper mucosal layers may give rise to kill-the-winner dynamics due to the lower levels of bacterial colonization and low VMR. This model is also supported by reports that rapidly evolving Ig-like domains expressed on phage capsids interact with mammalian host mucus glycans. This in turn results in subdiffusive motion of phage within mucus and allows them to persist in mucosal layers of the gut (86, 87).

**Figure 2:**
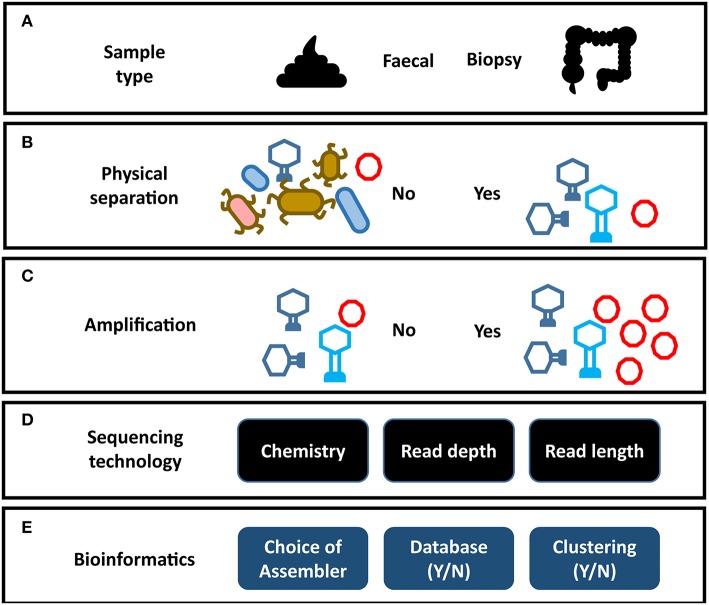
Impact of analysis choices on virome composition. Each step of a virome analysis protocol presents different options, each of which may affect the final outcome. **(A)** Sample type. **(B)** Physical separation of VLPs. **(C)** Amplification of virome DNA can preferentially amplify certain viral taxa (see Figure 3). **(D)** Sequencing chemistry, depth of sequencing and read length. **(E)** Assembly programs vary significantly in their ability to assemble virome data (see Figure 3). Reporting on the composition of viral sequences with homology to reference databases excludes the unknown majority of the virome. Clustering viral sequences by gene composition offers a promising alternative to database dependent methods by addressing high levels of sequence divergence in viral genomes.

While these models assist in our understanding of how the virome interacts with the microbiome and the gut environment, it is also important to consider their limitations. Phage-host dynamics can be expected to change both radially and longitudinally within the GIT (88) to reflect physical separation of phages and hosts (Figure 1C) and metabolic changes in the host populations (Figure 1D). They will also be heavily influenced by dietary components and the composition of the fecal matrix itself (5). To this end, sampling method and sample composition must be considered when drawing conclusions from virome data (Figure 2A). In the absence of studies of how these dynamics change along the human GIT, phage-host interactions must be interpreted as a snapshot of one particular point in space and time.

## Composition of the Gut Virome

The gut virome consists of two elements, the temperate phage located within bacterial genomes and the free virions or virus-like particles (VLP). The VLP fraction is obtained by applying several physical and enzymatic steps that remove dietary debris and prokaryotic and eukaryotic genetic material (Figure 2B) (29). As VLPs represent only a small fraction of the mass of the microbiome, virome studies that do not carry out this viral enrichment are limited primarily to the prophage sequences within bacterial cells (89, 90). However, these prophage sequences are in turn under-represented in studies that focus on the VLP fraction. In order to understand the virome as a whole, both elements need to be analyzed in tandem. The majority virome studies focus on one of these elements in isolation and as a result the apparent structure and composition of a virome is heavily dependent on the physical preparation of virome samples in the laboratory, the sequencing strategy, and the bioinformatic methodology employed (Figure 2). This is further complicated by the limited representation of human gut viruses in databases and a reliance on phage taxonomic classification systems which do not necessarily represent viral biology. Consequently, our understanding of the taxonomic composition of the phage populations in the human gut are predictably varied and contradictory.

### Taxonomic Composition

Sequence-based analysis suggests the identifiable fraction of the gut virome is dominated by small single-stranded DNA (ssDNA) phage of the *Microviridae* family and double-stranded DNA (dsDNA) phage of the order *Caudovirales* (26, 29, 30, 60, 91). However, bias in extraction methods has also been reported to skew the abundance of *Microviridae*, calling into question their dominance in the virome (discussed later) (92) (Figure 2C). The *Caudovirales* are classified into three families according to their distinctive virion morphology, consisting of a head and a tail structure making them easily distinguishable in microscopy studies. *Siphoviridae* exhibit long (sometimes up to 1 μm in length), non-contractile tails, *Podoviridae* exhibit short non-contractile tails, while *Myoviridae* exhibit a more rigid contractile tail composed of distinctive sheath proteins. These phage families have linear genomes, can encode a relatively large gene repertoire (in extreme cases containing over 600 genes) and can exhibit both temperate and lytic replication cycles. Early sequencing studies of the virome reported changes in *Caudovirales* community composition being associated with disease (25, 93, 94) and high *Caudovirales* abundance in EM images (28, 95). However, reported abundance in EM images could also be influenced by the fact that they are more easily identified than other viral taxa. Possibly as a result of these observations, *Caudovirales* composition and abundance has become a regular focus of virome studies, where it is used as a proxy for virome composition as a whole. However, as known *Caudovirales* tend to represent a small minority of VLP sequences in the human gut, caution should be taken when interpreting these results.

Due to the current structure-based classification system of *Caudovirales*, phage that exhibit significant functional similarities can be considered members of different families due to tail morphology. For example, phages P22 and lambda are classified as *Podoviridae* and *Siphoviridae* respectively, despite both undergoing the same replication cycle, sharing significant similarity in gene sequences and exhibiting identical genome organization (i.e., gene order and layout and regulation of transcription). Additionally this classification system is not grounded in sequence or protein sharing, yet sequence and protein homology are the primary methods of identifying *Caudovirales* in viromes (27, 34, 96, 97). This classification system is therefore limited in its ability to reflect the biological role or interactions of sequences classified as *Caudovirales*. A number of recent studies have also highlighted anomalies in *Caudovirales* taxonomy (98–100) and have proposed novel sequence-based methods to restructure the viral order (99). However, these are not without their own challenges, as shared genes and gene cassettes have been found to blur the boundaries between various dsDNA viruses (101, 102), which will be discussed in detail later.

### Case Study of the Most Dominant Gut Phage, CrAss

A prime example of the limitations of focusing on identifiable *Caudovirales* alone when carrying out virome analysis is that of crAssphage, one of the most abundant and successful biological entities of the mammalian gut. First reported in 2014 using novel assembly methods (103, 104), crAss was found to be six times more abundant in publically available gut metagenome samples than all other phage together. It made up to 90% of VLP sequencing reads and 30% of whole metagenomes in certain individuals, yet did not share homology with known reference databases (104). As a result, crAss would have gone entirely unnoticed using database-dependent analysis alone. CrAss has since been found to be globally distributed, with strains reflecting geographic distribution of human populations (105). Intriguingly, the presence of distant relatives to crAss in primates may suggest that crAss has coevolved with humans for millions of years from ancestors shared with primates, to modern *Homo sapiens* (105). These results highlight the importance of the unknown majority of the virome in human health and why it is critical to analyze the virome as a whole. Given the proposed ubiquity and dominance of crAss in the human gut it is also possible that the abundance of *Caudovirales* reported by EM studies may in fact reflect members of the extended crass-like phage family, which at the time were unknown.

The characterization of crAss and studying its role in the gut microbiome has been hampered by a lack of database representation, unknown host range and until recently (106), unsuccessful attempts to form plaques on agar overlays. However, its progression from an unknown abundant phage sequence to a characterized dominant member of the gut virome provides a useful framework in building our understanding of viral dark matter. Its initial discovery was built upon by using sensitive protein homology searches to identify an extended family of crAss-like phage from human gut virome samples (107, 108). Although sequence similarity across family members was low, protein family-based clustering identified conserved capsid proteins and predicted crAss to encode a short tail similar to that of the *Podoviridae* family of *Caudovirales* (108). Subsequently, crAss has also been identified in patients with diarrhea and in Malawian infants (107). Additionally it was found to be shared across healthy individuals and was capable of stable engraftment in FMT treatments (109). However, the host range and mechanism behind crAss ubiquity and abundance remained unknown.

Through the use of enrichment-based techniques the host for one member of the crAss family was confirmed to be *Bacteroides intestinalis*, a finding previously suggested by co-abundance profiling (104, 106). This provided new opportunities to study the phage-host interactions of the most abundant phage family in the human gut and its role in the microbiome. The ability to culture crAss *in vitro* also led to the description of one of the most intriguing aspects of this phage, its ability to coexist with its host at high abundance. This has a profound impact on our understanding of how the virome shapes the microbiome, as it appears that in crAss-rich individuals the dominant phage populations do not restrict the growth and proliferation of their host. Furthermore, it is believed that crAss persistence and coexistence with its host is not due to crAss undergoing temperate replication, as it does not possess any of the genes typically associated with lysogeny (106). Additionally, crAss prophage sequences have never been observed in the numerous *Bacteroidales* genomes available in sequence databases. It is possible that crAss replicates in a pseudolysogenic manner, existing as an episome within the host cell (41), that it facilitates a chronic infection without killing the host cell (42), or that it can exist in an extracellular carrier state (110). The high proportion of resistant host cells in culture (1–2%) could also suggest that a host cell carrying crAss in a pseudolysogenic state could confer resistance from infection as in superinfection exclusion. However, experimental evidence suggests crAss replicates in a lytic manner and its abundance in the gut is maintained by transiently resistant hosts. As phase variant CPS and transient resistance have been found to be central in phage-host dynamics in other *Bacteroides* species (71), it is possible that these mechanisms are also central to the unusual phage-host dynamics observed in crAss both *in vitro* and *in vivo*. This theory is supported by the reversion over time of some resistant clones to sensitive states (106). Given the abundance of *Bacteroides* and crAss in the human gut, phenotypic heterogeneity across a host populations may be the central mechanism to support stable interactions between some lytic phage and their hosts in the microbiome.

Another possible explanation for sustained crAss-host proliferation is the recently proposed “Royal Family” ecological model (111). This suggests continuous kill-the-winner dynamics occur at a strain-level rather than at a species or genus-level. In this way, the abundance of both phage and host would appear to be stable as fluctuations would occur below the level of detection. Subsequently, detailed analysis of strain-level variation between crAss and *B. intestinalis* could provide insights into the importance of this model in the GIT. A similar study carried out by De Sordi et al. (112) supports the “royal family model” and gave intriguing insights into its mechanics. This study described a point mutation in the tail fiber gene of P10 which is associated with strain-level host expansion from *Escherichia coli* LF82 to *E*. *coli* MG1655. Interestingly, this strain-level host switching was only observed when phage and both hosts coevolved within the murine model and was not observed when the two strains were cultured separately. Subsequent experiments revealed that switching required the presence of an intermediate host *E. coli* MEc1. These observations suggest that crAss-host stability in the gut could be caused by a wide array of strain-level and phenotypic variation. However, as strain-level variation is difficult to observe *in vivo* and *in vitro*, novel analytical approaches may needed to reveal crAss phage-host dynamics in the GIT.

The proliferation of both phage and host as is seen in crAss-*B. intestinalis* dynamics could also suggest that the presence of phage could confer an ecological advantage to the host. This phenomenon has been regularly reported in the ocean, where phage infecting cyanobacteria were found to carry auxiliary metabolic genes encoding photosynthetic genes (22, 113). Similarly, phage-mediated transformation of the host has also been well-established in disease such as the lysogenic phage encoding shiga toxin (23) and cholera toxin (114). It is therefore highly likely that in the dense and diverse ecosystem of the gut, extensive horizontal transfer of genes between hosts is facilitated by phage infection. In this way the virome may play a crucial role in shaping the functional capacity of the microbiome. One report of the presence of significant numbers of antibiotic resistance genes in gut virome sequences (115) was later shown to have probably resulted from bacterial contamination and confirmed that examples of phage-encoded antibiotic resistance genes were rare (116). This could be due to the efficiency of phage replication and the fitness cost of carrying antibiotic resistance genes. Without the selective pressure of antibiotics, viruses that pay the metabolic cost of carrying antibiotic resistance genes could be outcompeted (116). Additionally, the selective evolutionary pressure of remaining infectious in the face of a constantly adapting host may outweigh the need to preserve the host from an antibiotic. The follow up study also highlighted the importance of using stringent alignment criteria and validating results when classifying sequences or proteins. Due to the extensive unknown fraction of the virome and the difficulty in benchmarking classification criteria, lenient cut-offs can often lead to false conclusions of virome composition and function (116–118).

It is tempting to propose that the piggyback-the-winner hypothesis could be extended by considering the possible fitness advantages of carrying a prophage over and above superinfection exclusion. Such advantages could include carrying a virulence factor or providing access to a novel nutrient source (119, 120). Prophage-encoded fitness advantages have been observed in a number of pathogenic bacteria. These include prophage-encoded toxins (23, 114), alteration of O antigens in *Salmonella* and *Shigella* (121, 122) and phage-encoded glycosyl transferase operons which drive *Salmonella* LPS diversity (123). Furthermore, the horizontal transfer of virulence factors through temperate phage was found to be increased in gut inflammation (124). Additionally, recent observations in *Staphylococcus aureus* prophage (125) have described the packaging of chromosomal host DNA in phage capsids through a mechanism deemed lateral transduction. This mechanism suggests that phage-mediated horizontal gene transfer occurs at much higher rates than previously thought and that it plays a role in disease. However, the extent to which lateral transduction mediates gene transfer in the gut microbiome remains unknown. Examining phage-encoded auxiliary metabolic genes and how they shape the functional capacity of the gut microbiome is hindered at a large-scale metagenomic level by the complexity of fecal samples themselves. Dietary components and the sheer abundance and diversity of bacterial cells in feces make it difficult to completely remove bacterial sequences from virome samples. Use of density gradients such as CsCl are reasonably effective at generating viral particles devoid of cellular contamination, but will introduce bias in favor of particular viral capsid types (126) and are not suited to large-scale projects due to their associated manual workload. As a result, background contamination exists in the vast majority of virome samples (29, 116, 117) making it difficult to determine if a gene is of viral or bacterial origin. This may be further complicated by potential gene transfer agents (GTAs) (127) in gut VLP samples. GTAs are defective phage virions that exclusively carry fragments of bacterial chromosomal DNA and are used by bacteria as a means of HGT. As a result sequences within GTAs are difficult to differentiate from background contamination. However, the presence and prevalence of GTAs in human gut microbiome remains to be seen. Overall, despite the evidence of phage shaping the functional capacity of host communities, it is challenging to determine the extent to which phage transfer genes relevant to human health within the microbiome.

### Controversy Surrounding the Core Virome

The widespread geographical distribution and stability of crAss-like phage supports the concept of a core human virome, which was initially proposed by Manrique et al. (26). This was in response to a growing body of evidence that a core microbiome played an important role in human health. The study proposed a core of 23 viral sequences, one of which being the original crAss genome, which were shared across more than 50% of samples from an independent cohort of 62 healthy individuals. However, these findings were in stark contrast to the well-established belief that the human gut virome is highly individual-specific at a sequence level (29, 34, 35, 128). This disparity is largely due to the criteria used to define the presence of a viral sequence in a sample. If a single sequencing read from an individual could align to a particular viral assembly, the assembly was deemed to be present. This lenient criteria does not account for the modular nature and extensive gene sharing that occurs across dsDNA viral genomes (99, 101, 129). Thus, it would not be possible to differentiate the true presence of a viral sequence in a sample from the presence of a shared gene between two unrelated phage. This in turn, would lead to an inflated number of viral sequences being shared across individuals. However, the concept of a core virome has received support from a recent study with adult monozygotic twins, in which 18 contigs were found to be present in all individuals (*n* = 42) (34). Here, more stringent read recruitment criteria were applied to differentiate shared genes from the true presence of a viral sequence in a sample. Interestingly more than half of the viral assemblies identified across all individuals were homologous to crAss. It should be noted however, that these assemblies may also represent fragments of the same phage genome or family.

In contrast to these findings and to the proposed global distribution of crAss phage in human populations, the compilation of a large-scale gut virome database called into question the existence of a core human gut virome at a sequence level (24). By examining VLP metagenomes from 572 individuals, this study proposed that a core human gut virome does not exist. Recently, Shkoporov et al. (128) too made observations which support these findings by examining the virome of 10 individuals across a 12-month period. Here, a personal persistent virome (PPV) was observed that was composed of viral sequences detected in at least six of the 12 monthly time points. In accordance with previous longitudinal studies (60, 82), some viral sequences were present at nearly all time points within an individual. However, the virome was also highly individual-specific and viral sequences were not shared across the PPV of all individuals which supports the findings of Gregory et al. (24). This high level of inter-individuality in the gut virome hampers our understanding of the virome in disease as it is difficult to detect common viral signals within or between cohorts. While it is likely that the individuality of the virome is driven by infection and resistance dynamics, the level of taxonomic resolution at which the virome is studied is also a contributory factor (35). Sequence-based virome studies are carried out at the level of metagenomic assembly due to the absence of universal marker genes, limited database representation and established taxonomic organization. This represents species or strain-level resolution and is in contrast to the majority of bacterial metagenomics studies, which tend to be analyzed at higher taxonomic ranks such as genus or family. It is possible to find patterns across virome cohorts using a minor subset of known viral sequences and by excluding unknown sequences (25, 27, 130, 131). However, it is not known if these subsets represent the dynamics of the virome as a whole. To this end, a number of clustering programs have been developed that group viral sequences based on shared protein families (99, 129) such as vConTACT (99). This is a similar approach to that which was used to establish the extended crAss family despite low levels of nucleotide similarity between family members (107, 108). In this way, protein-based clustering of the virome can reveal compositional patterns across individuals that were not visible at a nucleotide level. Furthermore, this approach allows for both the known and unknown components of the virome to be included in analysis giving new perspectives to the virome in health and disease.

By applying this protein family clustering approach (102) to the same longitudinal cohort Shkoporov et al. (128) gained new insights into the existence and composition of a core gut virome across individuals. This phylogenetic core was composed primarily of crAss and *Microviridae* and was not identifiable at a nucleotide level. Intriguingly, this core was not composed of temperate phage which is in contrast to observations in previous reports (82). Furthermore, temperate phage were found to make up a minor subset of the core virome both within and between individuals. These findings suggest that mechanisms other than lysogenic replication are responsible for long term stability of the virome within healthy individuals. Moreover, they are in accordance with the global distribution and persistence of crAss in the human gut and ecological models such as the “royal family model.” Upon clustering the stable fraction of individual viromes (PPV) the largest and most interconnected viral cluster as associated with known *Caudovirales* sequences. This is in accordance with previous observations (99, 129) and reflects extensive shared genetic content across this order. It is also likely that this extensive gene sharing influenced previous database-dependent reports of temperate phage and *Caudovirales* dominance in the human gut virome. Furthermore, it highlights the importance of considering shared genes and gene cassettes when setting alignment criteria.

## Gut Virome in Disease

Given the extensive evidence that phage can shape the composition and function of bacterial communities, the virome of the human gut has been studied in a number of diseases. However, as with the concept of the core virome, findings have been somewhat contradictory and any potential role for the virome in shaping the microbiome in disease remains elusive. Studies have reported gut phage populations were not significantly altered in diseases such as colorectal cancer and HIV-associated AIDS (130, 132), despite established associations between the gut microbiome and these diseases (15, 133). This contradicts the established view that the gut virome and gut microbiome are closely linked and is more likely to reflect limitations of different analysis methods. These limitations include lenient alignment criteria to reference databases and the exclusion of viral dark matter from analysis. Furthermore, reports of changes in phage populations associated with diseases are often limited to changes in the composition of known *Caudovirales* (25, 90, 97, 131). Given the limitations of *Caudovirales* taxonomy and the challenges presented by extensive gene sharing across the order, these findings provide little insight into any role of the virome in disease.

An intervention study by Gogokhia et al. (134) sought to target cancer associated bacteria adherent invasive *E. coli* and *Fusobacterium nucleatum* with lytic phage in a germ-free mouse model. However, a direct interaction between the mammalian immune system and phage virions resulted in an exacerbated colitic reaction. The authors also proposed that phage DNA plays a central role in phage interaction with the mammalian immune system as empty phage capsids did not induce an immune response. Similarly an *in vitro* study using *S. aureus* and *Pseudomonas aeruginosa* phage observed a production of both pro and anti-inflammatory cytokines from peripheral blood mononuclear cells following endocytosis of purified phage virions (135). These observations are supported by the proposed ability of phage virions to cross the mammalian epithelial barrier *in vitro* via peptide sequences expressed on the capsid surface (136, 137). In this way it is possible that phage communities in the human gut shape the gut microbiome indirectly through interactions with the mammalian immune system. This concept of phage translocation and interaction with mammalian immune system has also been discussed in a number of reviews and perspective pieces as follows (138–141). Through the induction of a pro or anti-inflammatory response, phage could facilitate conditions that would favor a particular host or replication cycle. It is also possible that proposed phage–immune system interactions are driven by bacterial populations to facilitate infection or persistence in the human body. This was first demonstrated by lysogenic Pf phage which triggered maladaptive viral pattern recognition receptors and facilitated the chronic infection of *Pseudomonas aeruginosa* in murine and human cells (142). This was also the first reported case of a directly pathogenic effect of phage in bacterial infection and demonstrated that phage do not need to directly encode virulence factors to impact the virulence of their host.

### Fecal Microbiota Transplantation

FMT (fecal microbiota transplantation) is an emerging and experimental therapy that aims to restore healthy gut function through infusion of a fecal slurry from a healthy individual to the colon, cecum, or duodenum of the recipient. It has been shown to be very effective in the treatment of recurrent CDI with donor bacteria colonizing recipients for up to a year (143) and reported success rates of 80–90% (144). The first evidence that the virome had potential as a tool to shape the microbiome and may play a role in the efficacy of FMT treatment was reported by Ott et al. (145). In this study, patients with relapsing CDI received fecal filtrates from healthy donors that resulted in CDI symptoms being eliminated for up to 6 months. Furthermore, recipient phage populations were substantially altered, resembling those of the donor for a minimum of 6 weeks. Surprisingly, *Lactococcus* phage were reported to dominate both donor and recipient virome, despite *Lactococcus* spp. representing only a minor fraction of the gut microbiome. This could reflect a dominance of lactococcal phage in the donor and recipient, implying lactococcal phage play an important role in homeostasis in CDI. However, phage sequence databases are dominated by those which are industrially relevant and cultivable, which includes lactococcal phage (146). As a result, unknown sequences are statistically more likely to align to lactococcal phage and other cultivable or industrially relevant sequences when lenient alignment criteria are used. Thus, the dominance of lactococcal phage in the virome of FFT recipients is likely to be yet another artifact of database-dependent analysis methods. It should also be noted that, in accordance with the majority of database dependent virome studies, lactococcal phage are members of the order *Caudovirales*. This supports the concept that *Caudovirales* dominance reported at an order and family level in the gut virome could be driven by gene modules shared with *Caudovirales* in reference databases. However, the resemblance of the donor virome to that of the recipient suggests that, regardless of classification limitations, the virome plays a significant role in the maintenance of homeostasis in the gut. A subsequent study by Draper et al. (109) also reported the stable engraftment of donor phage populations in recipients for up to 12 months. In accordance with observations at a bacterial level (143, 147) successful phage engraftment was also dependent on specific donor-recipient pairings. It should be noted that the role of other elements present in the filtrate (i.e., chromosomal DNA, plasmids, bacterial cellular components, and signaling molecules) remains unknown and could also play a role in the restoration of healthy gut function in CDI. With conditions such as ulcerative colitis (UC) that are characterized by more subtle microbiome changes than CDI, successful FMT treatment (i.e., remission and mucosal healing) was not associated with changes in the phage population (148). This is in contrast to reports of alterations in bacterial diversity following FMT in UC (149). Additionally, changes in the diversity of phage populations between healthy and UC cohorts were not observed (148). These observations were in contrast with previous IBD virome studies, which reported differences in phage alpha diversity between healthy and UC cohorts (25, 97).

### Inflammatory Bowel Disease

Inflammatory bowel disease is a prevalent chronic disorder of the gastrointestinal tract with both genetic and environmental risk factors (150). The composition of gut bacteria and their interaction with the host immune system are believed to be central to its pathology, yet the etiology of the disease remains poorly understood. Given the evidence that the virome can interact directly with the host immune system and shape the composition and function of the microbiome, both fecal and mucosal phage communities have been studied in IBD. Current understanding of phage populations in IBD has focused on VLPs, proposing that disease-specific patterns of *Caudovirales* are linked to Crohn's disease (CD) and ulcerative colitis (UC). Furthermore, these changes in VLP composition have been reported not to reflect alterations of the bacterial community. However, the details of these compositional changes vary between studies (25, 27, 93–95, 151).

Early IBD virome studies using the Roche 454 sequencing platform were limited by sequencing depth. These studies observed lower diversity and greater variation in the fecal VLP communities of patients with CD relative to healthy samples (93). The same group performed another sequence based analysis of the fecal and mucosal VLP community of CD and observed greater viral load and diversity in the feces than mucosa of all individuals (151). Additionally, virome alpha diversity was reported to be significantly lower in disease. However, in contrast to their previous study, it was also reported that both healthy and CD cohorts were dominated by *Microviridae* rather than *Caudovirales*. Another study that analyzed the first mucosal virome in pediatric CD (94) proposed a dominance of *Caudovirales* phage overall and detected a single viral sequence in colonic mucosal samples from patients with CD. While this may suggest an extreme dominance of this virus in the mucosa of pediatric CD it more likely reflects insufficient sequencing depth resulting from low biomass samples and should be treated with caution.

As sequencing technology progressed, researchers were granted more detailed insights into the virome in IBD. However, these insights contradicted previous findings and highlight the impact of sequencing platform on results. This could also suggest that our understanding of the virome in disease will continue to change as sequencing technology progresses. Illumina-based studies reported disease-specific increases in *Caudovirales* alpha diversity in the VLP viromes of UC and CD compared to healthy controls in adults (25) and children (97). Intriguingly, these alterations were also reported not to reflect changes in the bacterial community (bacteriome). Conversely, decreased alpha diversity was observed in *Caudovirales* families from the mucosal VLP virome of UC relative to healthy controls (27). This supports the idea that viral communities and ecological models differ at different spatial locations within the gut. Additionally, it suggests that phage of the order *Caudovirales* play a central role shaping the microbiome in IBD. However, as with other studies of the virome in disease, these findings are limited to minor identifiable fractions of the dataset and offer little by way of insight into the role of phage in IBD.

In an effort to expand our understanding of the gut virome in IBD and move beyond the limitations of viral sequence databases, Clooney et al. applied the whole-virome analysis (WVA) (35) protocol discussed earlier to the keystone IBD virome dataset published by Norman et al. (25, 35). In this way, it was possible to gain novel insights into the composition viral dark matter in this disease. Contrary to the original findings of the study, alterations in the whole virome mirrored those of the bacteriome, and differences in overall virome alpha diversity were not seen across the whole-virome. In accordance with current understanding the gut virome, high levels of inter-individuality were observed, and were likely to conceal any patterns in virome composition across individuals. Subsequently, we followed the protocol established in Shkoporov et al. (128) to cluster viral sequences according to gene content. This revealed a core of primarily lytic phage in healthy individuals and supported observations of Shkoporov et al. (128). However, this healthy core was also found to be absent in patients with IBD where it appeared to be replaced by a community of temperate phage. The majority (six) of the eight viral clusters which made up the healthy core virome in this analysis did not share homology to known viral sequences further highlighting the biological signals that may be missed when relying on database-dependent methods. Interestingly, one of these healthy core virome clusters was identified as crAss. This supports previous observations of its ubiquity in healthy human populations, low rates of detection in unhealthy individuals and its role in the core healthy human virome.

One possibility is that in the inflamed gut, environmental stresses from the human immune response such as antibodies and reactive oxygen species, leads to increased induction of the prophage present in the bacterial microbiome. The physiology of bacterial cells and the composition of the bacterial community influences whether integrated prophage enter the lytic or lysogenic replication cycle (Figure 1A) (83, 152). It is therefore likely that temperate virome would react to environmental stress and resulting changes in the host community. This increased lytic replication and subsequent death of bacterial hosts would correspond with the observed reduction in bacterial alpha diversity associated with IBD. It would also correspond with the observed increase in in free phage virions (95) and temperate VLPs in disease (35). Additionally, the resulting increase of bacterial cell wall components and debris available to interact with the human immune system could perpetuate an inflammatory response. In accordance with *in vitro* reports, it is also possible that increased cell wall permeability associated with inflammation allows for increased phage translocation (137) and interaction with the host immune system (134, 135, 153). The observations of Clooney et al. (35) provide a novel theoretical, mechanistic rationale for the interaction of the whole virome and the microbiome in disease, beyond taxonomic assignment and compositional patterns of the known minority. Additionally, they provide comprehensive evidence to support the mechanisms that had been previously proposed by Norman et al. (25).

The extent to which the switch from temperate to lytic replication cycles and the composition of the core virome shape the gut microbiome and influence human health and disease remains speculative. However, the analysis approach outlined by Clooney et al. (35) paves the way to a better understanding of how the interplay between the microbiome and the virome reflects or influences human health and disease. By allowing the detection of biological signals across the entire virome it is now possible to identify viral signals associated with disease which had been previously undetected. The WVA approach is also supported by the methods used to characterize the crAss-like family (106–108). CrAss has progressed from an unknown viral sequencing anomaly to providing insights into the composition and function of the healthy human gut virome. In turn, the methods used in this progression provide a framework to characterize unknown but biologically relevant sequences identified by WVA.

It should be noted, that although many virome studies tend to report the dominance and composition of known *Caudovirales*, this provides little insight into the biological impact of these phages or how they shape the gut ecosystem. *Caudovirales* dominate reference databases, exhibit extensive gene sharing across families and orders and feature temperate phage genera (101, 102, 154). It is therefore likely that database-dependent methods of virome analysis classify unknown dsDNA virus sequences as known *Caudovirales* due to shared genes or gene cassettes and lenient detection criteria. As the representation of gut virome sequences in databases improves with efforts such as those carried out by Gregory et al. (24) and the characterization of crAssphage (106, 107), database-dependent analysis methods may be able to better reflect the composition and dynamics of the virome. However, as extensive gene sharing remains a central part of dsDNA viral genomes it is essential that stringent alignment criteria are used to differentiate shared functional modules from the presence of a particular virus (36, 155), regardless of the database used. Additionally, it is crucial that these alignment methods and their findings are validated to avoid misleading conclusions as to virome composition or function (116–118, 145).

## Addressing the Current Challenges of Virome Research

**Figure 3:**
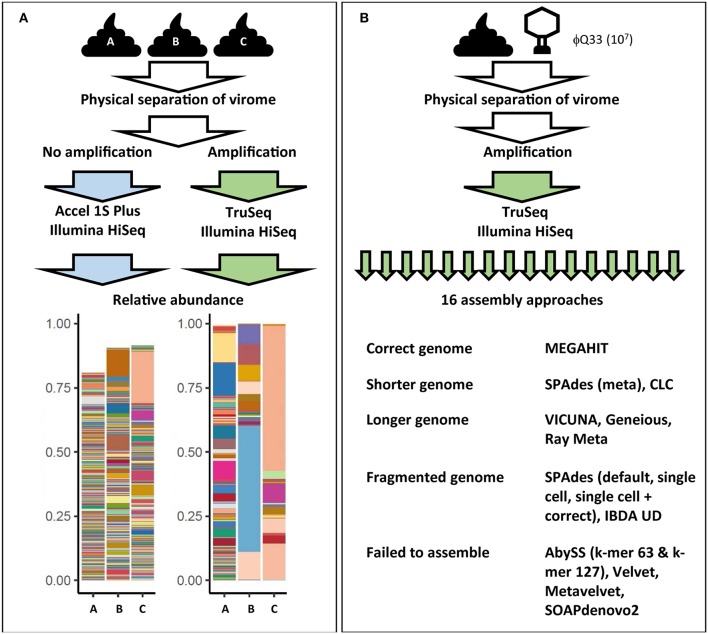
Examples of how virome composition is influenced by key steps in analysis. **(A)** Three samples were subjected to identical filtration and DNA extraction steps. One set was amplified and prepared for sequencing using the Illumina TruSeq library kit while another set of unamplified samples were prepared using the Accel 1S Plus kit. Both sets were sequenced on the Illumina HiSeq platform. Differently treated samples differ in terms of final composition, represented in bar plots. Each color represents the relative abundance of a unique viral contig in each sample. Abundance does not reach 100% in the unamplified sample as the higher level of richness also hampered assembly [adapted from (128)]. **(B)** Impact of assembly software on final virome composition. A fecal samples was spiked with φQ33 10^7^ PFU ml^−1^, extracted and sequenced. These sequences were assembled using 16 assembly programs. Only one assembler identified the genome in a single contig of the correct length. Five assemblers completely failed to assemble the genome and a further five generated fragmented assemblies (38).

As has been discussed, studying phage of the human gut microbiome is made challenging by the composition of the sample (usually a fecal specimen; Figure 2A). In order to enrich for the VLP fraction of the virome, extensive mechanical, chemical, and enzymatic processing is required to remove cellular DNA and dietary components (Figure 2B) (29, 126). Unfortunately, this results in particularly low DNA yields that can complicate the generation of sequencing libraries. This challenge is more pronounced in mucosal virome studies where DNA yields are lower again (132, 156). As a result, all but one (26) virome study to date have depended on multiple displacement amplification (MDA) of viral DNA to reach sufficient quantities to sequence. As with all metagenomics, it is crucial to find the balance between sequencing chemistry, depth of sequencing and read length. These factors have profound impacts on the final virome sequences available for downstream analysis. This was highlighted by the differences in virome alpha diversity reported by 454 pyrosequencing, when compared to deeper sequencing on the Illumina platform as previously discussed. Short read platforms such as the Illumina HiSeq are a means to perform deep sequencing of the virome with low error rates and relatively low input DNA requirements. However, these libraries can also lead to fragmented assemblies and poor recovery of viral genomes (38). Long read sequencing offers a promising solution to this assembly challenge, as it is possible to sequence entire viral genomes on a single read (Supplementary Table 1). This overcomes hypervariable sequences and repeat regions in viral genomes which hamper assembly (61). Currently long read sequencing platforms also require very large quantities of unfragmented DNA, which can be challenging acquire from virome samples. As a result, the initial DNA yield and the amplification step directly influence the sequencing chemistry, read depth and read length which can be used with virome sequences (Figure 2D). The MDA step has also been reported to introduce considerable bias into the composition of the resulting virome which must be considered when drawing conclusions from data (Figure 3A) (157, 158). Studies have also reported MDA preferentially amplifies small circular ssDNA viruses, which include the family *Microviridae* (Figure 2C) (92). This could call into question both the reported abundance and ubiquity of this family across individuals. Although it is difficult to quantify the extent to which preferential amplification occurs, recent meta-analysis of gut virome studies suggested *Microviridae* may be 10-fold lower in abundance than previously thought (24). It is believed that priming biases of the random hexamers used in the MDA reaction do not prime equally across all genomes, making quantitative interpretation of virome data difficult.

MDA bias can also have a significant impact on qualitative analysis of the gut virome. As the concentration of the DNA template also impacts the products of an MDA reaction, initial log-fold differences in the abundance of viral sequences are exaggerated by MDA (159, 160). This results in extremes of both high and low read coverage and uneven representation of the initial metagenome. As high-abundance sequences sequester sequencing resources, low-abundance sequences can be insufficiently covered (161). These coverage extremes have profound impacts on a number of steps in bioinformatic pipelines, but in particular metagenomic assembly (38, 161). A recent comparison of all assemblers used in virome studies to date observed that both high and low-coverage sequences resulted in fragmented assemblies and recovered only small proportions of viral genomes (38). Furthermore, samples were spiked with an abundant (10^7^ plaque-forming units ml^−1^) exogenous lactococcal phage Q33. These samples underwent identical extraction amplification and sequencing and resulting viromes were then assembled using all assembly methods which had been reported in virome studies at the time (16). Ten of the assemblers either failed to recover or significantly fragmented the Q33 genome and only one assembler recovered the genome at the correct length (Figure 3B) (38). These results suggest that numerous and potentially biologically relevant viral sequences may be not only be skewed in abundance but also excluded by current virome analysis protocols. This also means that we see the virome through the lens of the extraction protocol before any decision has been made to use database-dependent or independent methods (Figure 2, Supplementary Table 1).

As with many microbiome studies, conclusions from virome studies are primarily drawn from relative rather than absolute abundances of sequences. As has been discussed, these abundances are often skewed by MDA bias. This ambiguity highlights the need for quantitative analysis protocols in virome studies as was recently described (29, 35, 128). These studies reported significant differences in the overall viral load between individuals. This total viral load was also correlated negatively with viral alpha diversity within the sample and the presence of abundant and typically virulent phage such as *Microviridae* and crAss. These results suggest that high viral load is associated with a low number of abundant phage, which consequently mask underlying temperate phage diversity. This also suggests the maintenance of high-abundance non-temperate phage may be closely linked to the health status of the gut. Subsequently, this may act as a useful biomarker for disease and provide insight into the phage-host dynamics related to microbiome stability and disease status.

## Future Prospects and Conclusions

Sequence-based analysis of the bacteriophage in the human gut has revolutionized our view of the gut virome and its relationship with the microbiome. However, this new insight has also revealed how little we know about this relationship. Our current understanding is founded predominantly on extending knowledge generated in reductionist phage-host studies *in vitro*, or by large scale metagenomic studies of the VLP fraction. While these *in vitro* studies give detailed insights into the mechanics of individual phage-host interactions, the prevalence of these interactions in the gut ecosystem remains speculative. Additionally, numerous studies have also reported that these interactions can change dramatically in the gut (112, 134). Large-scale metagenomic studies suggest that the virome and the microbiome are closely linked, but these studies tend to give broad overviews of subsets of the virome and lack details on the phage-host interactions. This leads to significant gaps in our understanding of how phage-host dynamics *in vitro* differ from phage-host dynamics occurring in the gut.

Application of similar analysis methods across studies (i.e., MDA, alignment to reference databases, reports on *Caudovirales* alpha diversity) allows for comparison across samples and studies. However, many virome studies present inconclusive or contradictory results that hinder the progression of the field. Arguably the overreliance on these analysis methods is largely to blame for the gaps in our understanding of the virome. This is also supported by recent observations that methodology had greater impact on the conclusions drawn from virome studies than health or disease status (24). As conclusions are drawn from minor fractions of data and as detection criteria do not take into account phage biology and evolutionary history, we must pose the question: is the gut virome field built on unstable foundations? With this in mind, due caution must be used when interpreting the findings of virome data. As virome data is particularly sensitive to methodological bias, conclusions must be considered in the context of the analysis methods used (Figure 2, Supplemetary Table 1). These limitations highlight the need for radically new approaches to studying the virome if we are to understand its role in shaping the microbiome in health and disease.

Developments in sequencing library kits such as the Swift Biosciences Accel 1S Plus kit or extraction protocols like the linker amplified displacement LADs (36), offer potential solutions to creating unbiased sequencing libraries from low-input DNA yields. Through the removal of MDA bias and spiking known concentrations of exogenous phage (29), it may be possible to gain new insights into the true composition and absolute abundance of the virome. As has been discussed at length, the sensitivity of virome data to methodological bias highlights the critical need for extensive optimization and validation of all steps of virome analysis protocols, from wet-lab extraction protocols (29, 36, 155) to bioinformatic pipelines (37, 38, 161, 162). However, standardization and consistency must not be at the cost of developing new methods. Furthermore, when characterizing viral sequences it is crucial to use stringent detection criteria to minimize the impact of spurious alignments and the influence of gene sharing across dsDNA viruses.

Significant progress has been made in increasing the representation of gut phage in reference databases and there is a growing consensus that viral taxonomy will soon move toward sequence-based taxonomy (24, 163–166). However, proposed protocols to add metagenomic sequences to current taxonomic systems have not yet been accepted (167). Given the dominance of unknown sequences in virome data it is therefore crucial to accept the current limitations of phage taxonomy. Rather than force the virome to fit current taxonomic systems, we propose that future studies should allow the virome to reveal its own targets for downstream characterization. Subsequently, we have highlighted a method to analyze the virome in its entirety using the WVA protocol. Furthermore, we have described a framework to characterize unknown but biologically relevant viral sequences that may be identified using WVA. In this way it may be possible to address the gaps in our understanding of phage-host dynamics in the human gut, and see existing datasets in a new light.

To what extent the phage of the human gut shape the microbiome will dictate whether it will be possible to use phage as a therapeutic tool in the future. There are significant gaps in our understanding of phage-host interactions which need to be addressed before we can reach any conclusions on the usefulness of phage as a biotherapeutic. By increasing our understanding of phage-host interactions in the gut, it may be possible to pave the way for therapeutic applications of phage in the human body. However, the limited insights we have been granted to date of phage-host interactions have also highlighted some significant hurdles facing phage therapy. Early evidence that the virome could play a role in the success of FMT (109, 145) suggests there may be a future in using the virome to shape the microbiome in disease. To date, the majority of phage intervention studies are based on single phage-host pairs (or cocktails containing limited numbers of phage) *in vitro* which have been shown to be significantly different to phage-host dynamics *in vivo* (112). For example, phage have been found to switch hosts and interact directly with mammalian immune cells *in vivo*, which has serious implications for the future of phage therapy (134, 153). Additionally the phage-mediated transfer of host virulence factors (23, 114, 168, 169) as well as direct pathogenesis of phage capsids (142) suggests phage could be a potential risk in therapeutic settings. These challenges are confounded with regulatory issues (170) and additional gaps in our understanding of the pharmacodynamics of phage in mammalian tissue. Having the potential to directly interact with the immune system (86, 87, 136, 137) given their larger size relative to other biological therapeutic agents makes phage a more complex therapeutic agent than any that have preceded them. However, in light of the increasing incidence of bacterial pathogens which are resistant to antibiotics, and given the promising results of some existing phage therapy trials (171–174), overcoming these challenges is critically important. Similarly the gaps in our understanding of how phage shape bacterial communities will need to be addressed if phages are to have a role in avoiding future global health issues.

## Author Contributions

TS and CH drafted and approve of this manuscript.

### Conflict of Interest

The authors declare that the research was conducted in the absence of any commercial or financial relationships that could be construed as a potential conflict of interest.

## Supplementary Material

Table S1 Pros and Cons of key steps in virome analysis pipelines.
